# Draft Genome Sequence of *Enterobacter oligotrophicus* CCA3, Isolated from Leaf Soil

**DOI:** 10.1128/MRA.01289-20

**Published:** 2021-01-28

**Authors:** Hironaga Akita, Yuya Itoiri, Noriyo Takeda, Zen-ichiro Kimura, Hiroyuki Inoue, Akinori Matsushika

**Affiliations:** a Research Institute for Sustainable Chemistry, National Institute of Advanced Industrial Science and Technology, Higashi-Hiroshima, Hiroshima, Japan; b Department of Civil and Environmental Engineering, National Institute of Technology, Kure College, Kure, Hiroshima, Japan; c Graduate School of Integrated Sciences for Life, Hiroshima University, Higashi-Hiroshima, Hiroshima, Japan; University of Arizona

## Abstract

Enterobacter oligotrophicus CCA3 was isolated from leaf soil collected in Hiroshima Prefecture, Japan. Here, we report the draft genome sequence of E. oligotrophicus CCA3. The draft genome sequence of E. oligotrophicus CCA3 consists of 29 contigs of 4,425,100 bp, with a GC content of 54.2%.

## ANNOUNCEMENT

Oligotrophs can grow using poor nutrition, whereas growth is inhibited under nutrient-rich conditions. Consequently, oligotrophs have not been applied to industrial fermentation (1). If novel oligotrophs that are unaffected by nutrient-rich conditions are used for industrial fermentation, we expect that production costs could be reduced.

To isolate the desired oligotrophs, soil samples were suspended at 10% (wt/vol) in sterilized water. Each soil sample was filtered, and the filtrate was inoculated onto a 1.5% agar plate (2). After incubation for 1 day at 37°C, a single colony was obtained from the leaf soil filtrate. The purified strain was isolated by the dilution plating method and named strain CCA3 (strain number HUT-8140).

Strain CCA3 was pregrown at 37°C for 12 h in Nutrient Broth (Kyokuto). The precultures were inoculated into the fresh medium and cultured aerobically overnight. The cells were harvested by centrifugation and then washed twice with sterile water. After the genomic DNA was extracted using the illustra bacteria genomicPrep mini spin kit (GE Healthcare) at room temperature, 16S rRNA gene amplification and genome sequencing were immediately performed. The concentration and purity of genomic DNA were measured using a Quant-iT double-stranded DNA (dsDNA) broad-range (BR) assay kit (Invitrogen) and a NanoDrop ND-1000 spectrophotometer (Thermo Fisher Scientific), respectively. The 16S rRNA gene was amplified using KOD Plus (Toyobo) with the bacterial universal primers 27f and 1391r. The homology search was carried out using the NCBI BLAST program. The 16S rRNA gene sequence (GenBank accession number LC589703) of strain CCA3 showed 99.6% similarity to that of Enterobacter oligotrophicus CCA6^T^ (2).

The sequencing libraries were prepared using the Nextera XT DNA library preparation kit (Illumina), and genome sequencing was performed using a MiSeq sequencer (Illumina) with reagent kit v3 (Illumina). Default parameters were used for all software unless otherwise specified. *De novo* assembly of the raw data was carried out using A5-miseq v20160825 (3). tRNA and rRNA genes were detected using Prokka v1.14.0 (4). Genome annotation was performed using Prokka v1.14.0 (4). Average nucleotide identity (ANI) values were calculated using the ANI algorithm (5) implemented within the OrthoANIu tool (6). The raw data included 6,990,056 paired-end reads with a length of 301 bp that were used for the assembly. The genome sequence was 4,425,100 bp, with a GC content of 54.2% and coverage of 252-fold. The 29 contigs in the assembly yielded the largest contig of 860,849 bp and a contig *N*_50_ value of 384,860 bp. Within the genome sequence, 4,094 predicted coding sequences were identified, along with 86 tRNA genes and 16 rRNA genes. The ANI value analysis showed that strain CCA3 exhibited similarities of 99.9% to its closest relative, E. oligotrophicus CCA6^T^. These values exceeded the cutoff value of 98% for prokaryotic subspecies delineation (7, 8); therefore, strain CCA3 was identified as E. oligotrophicus CCA3.

### Data availability.

The draft genome sequence of E. oligotrophicus CCA3 has been deposited in the DDBJ/EMBL/GenBank databases under accession numbers BNJN01000001 to BNJN01000029. The raw sequence reads have been deposited in the DDBJ database under BioProject number PRJDB10684, BioSample number SAMD00253845, and DRA number DRA011084.
